# Hepatitis B surface antigen prevalence among 12 393 rural women of childbearing age in Hainan Province, China: a cross-sectional study

**DOI:** 10.1186/1743-422X-10-25

**Published:** 2013-01-18

**Authors:** Yu Zhang, Weiming Fang, Lichun Fan, Xiaohui Gao, Yan Guo, Wenming Huang, Yukai Du

**Affiliations:** 1 Department of Maternal and Child Health, School of Public Health, Tongji Medical College, Huazhong University of Science and Technology, 13th Hangkong Road, Wuhan, P.R, China; 2 Department of Prevention and Health Care, Hainan Maternal and Child Health Care Hospital, Haikou, China

**Keywords:** China, Women of childbearing age, Hepatitis B surface antigen, Seroprevalence, Risk factors

## Abstract

**Background:**

Hepatitis B virus (HBV) infection is highly endemic in China and it threats human health seriously. The hepatitis B surface antigen (HBsAg) prevalence among women of childbearing age plays an important role in mother to child transmission of HBV, as 30% ~50% of chronic carriers can be attributed to maternal-infantile transmission. However, there are few studies which have reported on the prevalence of HBsAg among women of childbearing age in China. This study aimed to determine the prevalence of HBsAg and its associated risk factors among rural women of childbearing age in Hainan, which is the highest hepatitis B virus endemic province in China.

**Methods:**

A cross-sectional, population-based study, which included 12393 rural women aged 15 ~ 49 years, enrolled by a multistage stratified cluster sampling, was carried out in Hainan province, China, from November 2007 to December 2008. Blood samples were obtained from each study participant, and screened for HBsAg.

**Results:**

The overall HBsAg prevalence of childbearing age women was 9.51%. Risk factors for HBsAg positivity among rural women were: lower education level (OR=1.206), lower family monthly income (OR=1.233), having an HBsAg-positive family member (OR=1.300), without an immunization history (OR=1.243), tattooing (OR=1.190), body piercing (OR=1.293), vaginoscopy history (OR=1.103) and history of induced abortion (OR=1.142).

**Conclusions:**

There is a high HBsAg seroprevalence rate among rural women of childbearing age in Hainan province. Hence, it is necessary to take preventive measures to reduce the seroprevalence of HBsAg and to control its associated risk factors.

## Background

Hepatitis B virus (HBV) infection is an important public health problem worldwide, more than two billion people (one third of the world’s population) have been infected with HBV, and between 350 and 400 million people have chronic liver infections with the presence of hepatitis B surface antigen (HBsAg) [1-3]. A range of 25% ~ 40% of patients with chronic HBV infection eventually develops the complications of cirrhosis and/or hepatocellular carcinoma (HCC) [4]. Globally, the prevalence of chronic HBV infection is classified into three groups: high prevalence (> 8%), intermediate prevalence (2% ~ 8%) and low prevalence (< 2%) [2,5]. China is classified as having a high endemicity of hepatitis B virus [1,6]. Furthermore, China is now the only country in Asia that remains in high HBV endemicity, with 7±20% prevalence of HBsAg positivity [6]. According to a national survey conducted in 1992, the overall prevalence of HBsAg in the Chinese population was 9.75%, and the HBV infection rate was as high as 50.04% [7], which were much higher than many other countries.

The first serologic marker of HBV infection is HBsAg, which can be detected from 2 to 12 weeks after infection with HBV. The presence of HBsAg indicates that the person is potentially infectious [8]. HBsAg positive mothers are believed to account not only for mother to child transmission at birth but also for the intrafamilial horizontal transmission of HBV during infancy and early childhood [9]. Vertical transmission is the major cause of HBV transmission in China. There are more than 130 million chronic carriers of HBV in China, 30% ~ 50% of which are attributed to maternal-infantile transmission [10]. Children born to HBsAg positive mothers have a significantly higher risk of becoming HBsAg positivity than those born to HBsAg negative mothers [11]. The prevalence of HBsAg among women of childbearing age plays a very important role in the transmission and prevalence of HBV. Although some studies have reported on the seroprevalence of HBsAg and its related risk factors among the general population [12,13], blood donors [14,15], pregnant women [16,17] and health care workers [18] in the past few years, few studies have focused on women of childbearing age (15 ~ 49 years).

Hainan province is the youngest province and biggest special economic zone in China, located in the southernmost part of China and surrounded by the sea. The results of the Chinese national HBV seroprevalence survey in 1992 has shown that Hainan was a highly hepatitis B virus endemic region, with the prevalence of HBV and HBsAg was 84.77% and 16.54%, respectively; both of them were much higher than other provinces in China [7]. This high prevalence of HBV infection seriously hindered the development of economy and the quality of people’s health in Hainan. The aim of present study is to explore the seroprevalence of HBsAg and its related risk factors among rural women of childbearing age in Hainan.

## Results

This study included 12 393 rural women of childbearing age with an age range of 15 to 49 years and a mean age of 32.25 ± 7.68 years, while the median age of 34 years. All of their serum samples were tested for the presence of HBsAg. A total of 1179 women tested positive for HBsAg. The prevalence of HBsAg positivity in this population was 9.51% (95% CI: 8.99 ~ 10.02). The mean age of HBsAg positive women was 35.39 ± 7.48 years and their median age was 35 years.

### HBsAg seroprevalence with different socio-demographic characteristics

Table 1 presents the seroprevalence of HBsAg with socio-demographic characteristics of the study samples. HBsAg carrier rate was significantly associated with age (*p*=0.001). The prevalence of HBsAg was 11.37% in the age group of 30 ~ 34 years, 10.51% in 25 ~ 29 years old, 10.32% in 20 ~ 24 years old, and 7.35% in 45 ~ 49 years old. Rural women of Li ethnic minority in Hainan had a higher level of HBsAg positivity than those of Han and other ethnic minorities (*p*=0.001). HBsAg positivity was seen to be higher among childbearing age women who were divorced or widowed (10.25%, 95% CI: 8.29 ~ 12.21) than single and married women, although there was no significant difference among different married status (*p*=0.393). Education level had a statistically significant effect on HBsAg positivity among women of childbearing age (*p*=0.002). Rural women of childbearing age who were housekeepers had a higher HBsAg positivity than whose who were employed (*p*=0.010).

**Table 1:** Seroprevalence of HBsAg with socio-demographic characteristics of the study participants

**Characteristic**	**Samples size (n)**	**Percent (%)**	**HBsAg positive (n)**	**Seroprevalence (%, 95% CI)**		***χ***^***2***^	***p***
**Age (y)**							
15-19	876	7.07	75	8.57	(6.71-10.42)	22.340	0.001
20-24	1353	10.92	140	10.32	(8.70-11.94)		
25-29	1783	14.39	187	10.51	(9.09-11.93)		
30-34	1754	14.15	199	11.37	(9.88-12.86)		
35-39	2174	17.54	206	9.48	(8.25-10.71)		
40-44	2408	19.43	222	9.21	(8.06-10.37)		
45-49	2045	16.50	150	7.35	(6.23-8.48)		
**Ethnic group**							
Han	6831	55.12	620	9.08	(8.40-9.76)	9.636	0.001
Li	4395	35.46	464	10.56	(9.65-11.47)		
Others	1167	9.42	95	8.14	(6.57-9.71)		
**Marital status**							
Single	4219	34.04	382	9.05	(8.18-9.92)	1.867	0.393
Married	7257	58.56	703	9.68	(9.00-10.36)		
Divorced/Widowed	917	7.40	94	10.25	(8.29-12.21)		
**Education level**							
Illiteracy	2813	22.70	309	10.98	(9.82-12.14)	14.442	0.002
Junior high	4421	35.67	427	9.66	(8.79-10.53)		
Senior high	3754	30.29	336	8.95	(8.04-9.86)		
College or higher	1405	11.34	107	7.62	(6.23-9.01)		
**Employment**							
Housekeeper	7314	59.02	737	10.08	(9.39-10.77)	6.574	0.010
Employee	5079	40.98	442	8.7	(7.92-9.48)		
**Family monthly income (RMB)**							
<1000	3931	31.72	411	10.46	(9.50-11.42)	10.816	0.013
1000~2000	4897	39.51	473	9.66	(8.83-10.49)		
2000~5000	2660	21.46	216	8.12	(7.08-9.16)		
≥5000	905	7.30	79	8.73	(6.89-10.57)		
**Nulliparous woman**							
No	5120	41.31	623	12.17	(11.27-13.07)	71.415	<0.001
Yes	7273	58.69	556	10.86	(10.14-11.58)		
**Location**							
East of Hainan	4012	32.37	336	8.37	(7.51-9.23)	9.010	0.011
Middle of Hainan	4123	33.27	411	9.97	(9.06-10.88)		
West of Hainan	4258	34.36	432	10.15	(9.24-11.06)		
**Total**	12393	100.00	1179	9.51	(8.99-10.02)		

The prevalence of HBsAg was higher among women with lower family income (<1000 RMB/ month) than in the group with higher family monthly income (*p*=0.013). In this study, 58.69% of women were nulliparous; the prevalence of HBsAg among nulliparous women (10.86%, 95% CI: 10.14 ~ 11.58) was significantly lower than among multigravidae (12.17%, 95% CI: 11.27 ~ 13.07) (*p*<0.001). When we compared the residential status and HBsAg positivity, the results showed that 432 (10.15%, 95% CI: 9.24 ~11.06) rural women who came from the west of Hainan province tested positive for HBsAg, significantly higher than those who came from the centre and east of Hainan (*p*=0.011).

### Risk factors of HBsAg seroprevalence among women of childbearing age

Univariate analysis by *Chi*-square test was carried out to identify factors associated with the prevalence of HBsAg. The independent variables included education level, family monthly income, have an HBsAg-positive family member, tattooing, body piercing, immunization history, history of dental surgery, history of surgery, transfusion history, vaginoscopy history, endoscopy history and history of induced abortion. As results showed in Table 2, there was no association between HBsAg prevalence and history of dental surgery, history of surgery, transfusion history and endoscopy history (*p*>0.05), but the rest of the variables increased risk of HBsAg. Rural women who had junior high school or lower education level had a higher prevalence of HBsAg than with a senior high school or higher education level (OR=1.206, 95% CI: 1.066 ~ 1.365). The HBsAg infection risk among women whose family monthly income were lower than two thousand RMB was higher than those with family income above two thousand RMB (OR=1.233, 95% CI: 1.075 ~ 1.416). Having an HBsAg-positive family member was also a risk factor of HBsAg infection (OR=1.300. 95% CI: 1.075 ~ 1.416). Individuals who reported no history of immunization had a higher prevalence of HBsAg than those with a history of immunization (OR=1.243, 95% CI: 1.096 ~ 1.410). There was a significant relationship between tattooing and body piercing with HBsAg seroprevalence (OR=1.190, 95% CI: 1.005 ~ 1.410; OR=1.293, 95% CI: 1.110 ~ 1.506, respectively). Furthermore, the HBsAg prevalence among women who had ever had a vaginoscopy or history of induced abortion were higher than those women who never had such experiences (OR=1.190, 95% CI: 1.005 ~ 1.410; OR=1.293, 95% CI: 1.110 ~ 1.506, respectively).

**Table 2:** Possible risk factors for HBsAg seroprevalence in rural women of childbearing age

**Variables**	**Percentage of total subjects, %**	**HBsAg Seroprevalence, %**	**OR**	**95% CI for OR**	***p***
**Education level**					
Junior high or lower	58.37	10.17	1.206	1.066-1.365	0.003
Senior high or higher	41.63	8.59	1.000		
**Family monthly income (RMB)**					
<2000	71.23	10.01	1.233	1.075-1.416	0.003
≥2000	28.77	8.27	1.000		
**Have an HBsAg-positive family member**					
Yes	14.61	11.10	1.300	1.102-1.535	0.002
No	64.30	8.76	1.000		
Unknown	21.09	10.71	1.250	1.080-1.447	0.003
**Immunization history**					
Yes	39.10	8.40	1.000		
No	61.90	10.23	1.243	1.096-1.410	0.001
**Tattooing**					
Yes	13.12	10.89	1.190	1.005-1.410	0.043
No	86.88	9.31	1.000		
**Body piercing**					
Yes	77.33	9.99	1.293	1.110-1.506	0.001
No	22.67	7.90	1.000		
**History of dental surgery**					
Yes	21.34	10.28	1.117	0.969-1.289	0.128
No	78.66	9.30	1.000		
**History of surgery**					
Yes	34.03	9.89	1.068	0.942-1.211	0.307
No	65.97	9.32	1.000		
**Transfusion history**					
Yes	8.75	11.07	1.205	0.986-1.472	0.067
No	91.25	9.36	1.000		
**Vaginoscope history**					
Yes	18.32	10.22	1.103	1.110-1.506	0.001
No	81.68	9.36	1.000		
**Endoscopy history **^**a**^					
Yes	12.92	10.12	1.082	0.909-1.289	0.376
No	87.08	9.42	1.000		
**Induced abortion history**					
Yes	37.53	10.23	1.142	1.010-1.290	0.034
No	62.47	9.08	1.000		

^a^ not including vaginoscopy history.

## Discussion

Hepatitis B has been a serious public health problem in Hainan for a long time. In 1992, the Chinese national HBV seroprevalence survey found that the HBsAg seroprevalence of the population in Hainan was 16.54%, ranking first in China [7]. The seroprevalence level of HBsAg of 9.51% in this study of the childbearing age women in Hainan is higher than the 7.18% found among the overall population of China [19]; it is also higher than those women of childbearing age in Jiangsu province, China (6.71%) [17], pregnant women in Catalonia, Spain (1.2%) [16] and in Greece (1.16%) [20]; but lower than women of childbearing age in Madagascar (13.6%) [21]. Overall, the high positive rate of HBsAg among rural women of childbearing age in Hainan highlights the need for public health workers and government to come up with preventive measures of reducing the prevalence of HBsAg among women of childbearing age.

This study showed that HBsAg carrier rate was significantly related with age of the rural women. The highest prevalence of HBsAg is in the age group of 30 ~ 34 years, followed by the age group of 25 ~ 29 and 20~24 years, which are at the peak period of pregnancy. Similar results were found in other studies conducted in Turkey [22], Lorestan, West of Iran [23], Nicobarese tribe [24] and Mexico which was a country of low endemicity for HBV [25]*.* Mother to child transmission is an important transmission route for HBV; 30% ~50% of chronic carriers of HBV in China can be attributed to maternal-infantile transmission [10]. Furthermore, Ranger-Rogez S [26] pointed out that if the mother was HBsAg positive, plus HBeAg and DNA positive, the risk for the neonate to be infected was about 90%, and this child would become a chronic carrier in 80%~90% case. The prevention and control of HBV infection among women of childbearing age should therefore, be a priority of public health intervention in order to reduce the transmission of HBV from mother to child.

Our study found that the HBsAg positivity rate among women of Li ethnic group was higher than Han and other ethnic groups. Li people are the largest ethnic minorities in Hainan, with 14.73% of all Hainan population. Li people are the aboriginal peoples of Hainan, with a history of thousands of years. There are 1.25 million people of Li ethnic in China, and 1.11 million of them are from Hainan. However, epidemiological data on the different prevalence rate of HBsAg between Li women and women from the other ethnic background in China are limited, only a few studies have been conducted in counties and cities of Hainan province [27,28]; and their results are consistent with this study. As regards to few epidemiological studies conducted on the subject among Li people, the cause of high prevalence rate of HBsAg among this group is still unknown. Despite this, there are two possible reasons. Firstly, Li people have a different dietary habit and lifestyle from Han people, for instance, communal eating and living together is commonly practiced among Li people [29]. An experiment done by Bancroft WH *et al.*[30], demonstrated that human saliva can serve as a vehicle for the transmission of hepatitis B virus. Another study has also shown that HBV may occur in households with a persistent carrier probably via saliva or open wounds, although it is less efficient than sexual or perinatal transmission [31]. Secondly, there may be HBV susceptibility genes among Li people; for ethnicity might play a role in the different prevalence rates of the HBsAg [32]. This calls for molecular epidemiological studies to verify this notion.

In this study, the HBsAg prevalence was strongly correlated with low education level and low family income. The prevalence of HBsAg in women with junior high or lower education level was higher than in those with higher level, which is consistent with previous studies conducted in general population samples [12,23]. People with a higher level of education tend to efficiently utilize health services like vaccination, health education, high quality dentistry services, etc. The prevalence of HBsAg in individuals with a lower family monthly income (<2000RMB) was significantly higher than those with a higher income level (≥2000RMB). This result is similar to other studies’ reported that positive HBsAg status was less likely to occur among people with high socioeconomic status compared to those with low or middle socioeconomic status [32,33]. Poor and crowded living conditions, which are common in rural areas, can facilitate horizontal transmission between siblings, especially with a high rate of HBsAg carriers, as described elsewhere [31]. The finding of our study indicated that HBsAg positivity rate among rural women of childbearing age in Hainan province was geographically influenced. The positivity rate tended to be higher in the western region followed by the central and the east region, which was opposite to the economic level distribution pattern [34]. This finding agrees with the finding in our study, that economic status was a major risk factor of HBsAg prevalence.

Another most common factor observed was having an HBsAg-positive family member. Previous researches found similar results deduced from the general population [13,29,35]. Our results demonstrated that no previous history of vaccination significantly increased risk of HBsAg infection. This illustrates the need and importance of expanding and re-intensifying immunization program with extension to adults.

Rural women of childbearing age with tattoo and body piercing were significantly higher than those with no tattoo or body piercing. In a study by Zhang *et al.*[36] in Canada, tattooing and body piercing increased the risk of HBsAg significantly. In Mexico with low endemicity for HBV also have found tattooing was risk factor detected with a high frequency [25]. But Komas *et al.*[37] described that tattooing or body piercing did not increase the risk. This difference might be due to the different ethnic study population. In our study, all the subjects were female, which may be biased, as females were more likely to pierce than men. Having a vaginoscopy history and a history of induced abortion among women of childbearing age were two critical risk factors for HBsAg positivity which were different from other population. The prevalence of HBsAg among women with a history of vaginoscopy and induced abortion was significantly higher than those without. We do not know whether this results from confounding bias or other reasons, since there are no related studies to support these findings. However, this illustrates the need to pay seriously attention to promote safe sex behaviours among women of childbearing age to maintain good health of the reproductive system.

In this study, marital status was not a related factor to HBsAg, although some studies have shown marriage and heterosexual relationship to be related to HBsAg positivity [12,38]. In a study by Roman *et al.*[25] in Mexico illuminated that marital status had denoted an OR of 1.92 in single or divorced pregnant women and 7.4 in widows among general population. These differences are probably due to cultural differences in each population. Like many other studies [12,24,37,39], this study showed that a history of blood transfusion, surgery, dental surgery and endoscopy was not a risk factor for HBsAg. Although a study by Zali *et al.*[35] reported that a history of major surgery was associated to HBV. This probably indicates that there is a good surgery and nursing care environment in Hainan.

This study had three limitations. First, our study was a cross sectional design which was therefore, difficult to establish causal relations. Nevertheless, the findings provide important demographic insights on HBsAg positivity among rural women of childbearing age in Hainan province. Another limitation is that HBV infection can be transmitted efficiently through sexual intercourse and the role of sexual behavior in HBsAg seropositivity was not assessed. Thirdly, this study did not include urban women, who happen to have different economic levels, living surroundings, medical environment and conditions and etcetera from rural population which has impact on HBsAg seroprevalence. Therefore, we plan to conduct a sero-epidemiological survey of urban women in Hainan in the future.

## Conclusion

HBsAg seropositivity among women of childbearing age is a threat not only to their health but the next generation who are at risk of HBV vertical transmission. The prevalence rate of HBsAg among women of childbearing age plays a very important role in the spread of HBV. This study shows that the overall HBsAg seroprevalence among rural women of childbearing age in Hainan was 9.51%, which was high. So efforts should be made to control and prevent the HBV infection among childbearing age women in Hainan with focus on the reported risk factors. Vaccination program should be implemented not only in infants and children but also in adults. Further, the routine maternal HBsAg screening should be promoted and strengthened, especially in rural areas, to the benefit of taking appropriate and effective interventions to prevent mother-to-child transmission of HBV.

## Methods

### Study setting and population

The present study is a cross-sectional, population-based study, carried out from November 2007 to December 2008 in Hainan province, an area which is encircled by the sea, located in the southernmost part of China. It is the youngest province and biggest special economic zone in China. Hainan has a female population of 4.284 million, and 27.08% (1.160 million) of them are rural women of childbearing age [34]. This survey covered all 18 counties and cities in Hainan. The 18 counties and cities were divided into three regions according to geographical location (i.e. the east, centre and the west). According to the Hainan Provincial Bureau of Statistics that the economic level of the eastern part of Hainan has the best economic development as compared to the west and central regions [34].

Healthy rural women aged 15 to 49 years who were permanent residents of Hainan were eligible for enrollment in the study. The study population was obtained from the list of native residents using a multistage stratified cluster sampling method. The first step was to stratify, all the cities and counties were stratified into three levels according to economic status and then two sites were randomly selected from each economic level. Using this selection method, six towns were selected from each city and county, and a total of 108 towns of Hainan were selected. Thereafter, we randomly selected three villages from each town and we ended up selecting 324 villages. We selected 30 percentage of women aged 15 to 49 years in each village. A total of 12393 rural women of childbearing age were randomly selected and invited to participate in this survey.

### Samples and data collection

A questionnaire which included age, ethnic group, education level, marital status, employment status, family monthly income, history of dental surgery, transfusion history, immunization history, endoscopy, tattooing and some other possible risk factors was completed by each participant before collecting the blood samples. Then 5 ml venous blood samples were obtained from each participant. All fresh serum samples were tested for the presence of HBsAg, using qualitative enzyme-linked immunosorbent assay (ELISA) kits (Yingkechuangxin Company, Xiamen, China). Other markers were not tested. The test was carried out and interpreted according to the manufacturer’s instructions.

### Statistical analysis

The database was constructed with Epidata 3.1. Statistical analysis were performed with SPSS 13.0 software for Windows (SPSS Inc., Chicago. IL). Proportions, ratios, and median were applied to describe the socio-demographic characteristics of participants and infection status of HBsAg. *Chi*-square test was used to identify independent risk factors associated with positive test of HBsAg infection, odds ratios (ORs) and their 95% CIs were also calculated. The level of statistical significance was set at *P* ≤ 0.05.

### Ethical approval

The study was approved by the Ethics Committee of the Medical Association of Tongji Medical College of Huazhong University of Science and Technology. A written informed consent form was obtained from each study participant before blood sample and data collection.

## Abbreviations

HBV: Hepatitis B virus; HBsAg: Hepatitis B surface antigen; HCC: Hepatocellular carcinoma; CI: Confidence interval; OR: Odds ratio.

## Competing interests

The authors declare that they have no competing interests.

## Authors’ contributions

YZ and WMF performed the statistical analysis and prepared the manuscript. YKD and LCF participated in the design and coordination of the study and revised the manuscript. XHG, WMH and YG collected the data and help to draft the manuscript. All authors read and approved the final manuscript.
